# Highlight on Engineering *Mycobacterium smegmatis* for testosterone production

**DOI:** 10.1111/1751-7915.12466

**Published:** 2016-12-05

**Authors:** Utkarsh Sood, Yogendra Singh, Mallikarjun Shakarad, Rup Lal

**Affiliations:** ^1^ Department of ZoologyUniversity of DelhiDelhi110007India

## Abstract

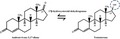

## Background

The development of male attributes such as external genitalia and secondary sexual characteristics is due to a hormone called testosterone produced in the testicles. This hormone is also responsible for the development and maintenance of muscle mass (Griggs *et al*., 1989), bone density, red blood cell counts (Bachman *et al*., 2014), supports sexual and reproductive function and contribute to a man's sense of anger and vitality (Batrinos, 2012).

While the underlying mechanism is not known, the production of testosterone in males gradually declines with age, beginning at around 30 (Morley and Perry, 2000). The decline is associated with a near‐total lack of interest in sex, erectile dysfunction (Kratzik *et al*., 2005), diabetes (Barrett‐Connor, 1992), Alzheimer's disease (Moffat *et al*., 2004), cardiac failure (Kontoleon *et al*., 2003), hypercholesterolaemia (*Haffner et al.,* 1993), osteoporosis (Campion and Maricic, 2003), frailty, obesity (Svartberg *et al*., 2004), hypertension (Phillips *et al*., 1993) and ischaemic heart disease (Barrett‐Connor and Khaw, 1988). The idea of supplementing declining levels of testosterone to treat diseases was realized by the scientific community as early in the mid‐1930s (Hamilton, 1937). In recent years, the use of testosterone therapy has become more widespread, but the only available synthetic form of testosterone is still expensive. Hence, a very little fraction of those affected are able to afford this therapy.

Microbes are ubiquitous and humans have exploited their biological processes, especially through genetic manipulation for the production of hormones and antibiotics. In 1978, genes encoding human insulin was cloned and expressed in *E. coli* followed by cloning of growth hormone in 1979, which replaced the unhuman forms of both these hormones. However, until now, testosterone (TS) has been chemically produced from androst‐4ene‐3,17‐dione (AD) (Ercoli and Ruggierii, 1953). Not only being expensive, the synthetic form has been reported to induce several side‐effects such as allergy, nausea or vomiting, impotency, painful or difficult urination, high levels of calcium in the blood, mild truncal acne, weight gain (Matsumoto, 1990) and coronary heart disease (Tripathy *et al*., 1998). In mammals, 17‐ketosteroid reductase (17β‐HSD) enzyme catalyses the synthesis of TS from AD. There was a hunt for microbial sources – both bacterial and fungal enzymes (Donova *et al*., 2005) that could convert AD to TS in order to reduce the production cost and benefit patients allergic to the synthetic derivative of TS. The ability of microorganisms to reduce 17‐keto‐ to 17β hydroxysteroids was first reported in *Saccharomyces cerevisiae*, during the transformation of androst‐4‐ene‐3,17‐dione to testosterone (Charney and Herzog, 1967). Subsequently, the ability to carry out 17β‐reduction of AD was reported for a variety of microorganisms of different taxonomy, including *Mycobacterium*,* Pediococcus, Brevibacterium, Bacillus, Arthrobacter, Lactobacillus* and *Nocardia (*Wix *et al*., 1968; Uwajima *et al*., 1973; Mahato and Mukherjee, 1984; Dutta *et al*., 1992; Kumar *et al*., 2001 *)*. But, very few microbial 17β‐OH SDHs were isolated and characterized. The most investigated enzyme is 3(17)β‐hydroxysteroid dehydrogenase (3(17) β‐OH SDH) isolated from *Comamonas testosteroni* (earlier classified as *Pseudomonas testosteroni*) (Groman and Engel, 1977). Attempts were made to introduce the 17β‐hydroxysteroid gene from *Comamonas testosteroni in E. coli* (known model system for biotechnological processes) (Plésiat *et al*., 1991). However, substantially low uptake of AD across the cellular membrane by *E. coli* limited the production process instigating researchers to look for novel biological models for the industrial production of testosterone. Considering the cost and the side‐effects, attempts have been made to produce testosterone *in vitro,* from AD using recombinant murine 17β‐HSD type V and glucose dehydrogenase as a cofactor (Fogal *et al*., 2013). However, this approach was not employed for industrial purposes due to the high cost of the production process.

A recent article by Fernández‐Cabezón *et al*., (2016) proposes a biological model for industrial production of testosterone using *Mycobacterium smegmatis*. This model overcome the first major bottleneck, that is uptake of AD by a bacterium. *M. smegmatis* can efficiently transport AD across its cellular membrane and does not degrade AD. Therefore, it was engineered for the biotransformation of AD to TS. For this, two genes – one from the bacterium *Comamonas testosteroni* and the second from the fungus *Cochliobolus lunatus* encoding microbial 17β‐hydroxysteroid:NADP 17‐oxidoreductase – were selected and introduced in the host cells. The host strains were *M. smegmatis* (wild‐type) and a genetic‐engineered androst‐4‐ene‐3,17‐dione (AD)‐producing mutant. First, Fernández‐Cabezón and co‐workers cloned both these genes in pMV261, an *E. coli*/*M. smegmatis* shuttle vector under the control of a constitutive promoter. This was then followed by cloning of these two genes in two different plasmids (pHSDCT and pHSDC) that were transformed in wild‐type and mutant *M. smegmatis* strain. The recombinant strains were able to produce TS from sterols or AD with high yield when compared with the production by mycobacterial strains obtained by conventional mutation procedures (Liu *et al*., 1994; Liu and Lo, 1997).

**Figure 1:**
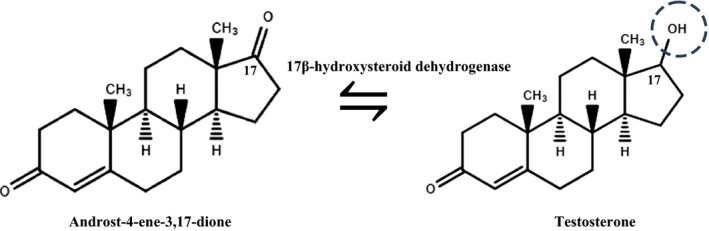
Hydrogenation reaction of Androst‐4ene‐3,17‐dione to testosterone by 17β‐hydroxysteroid dehydrogenase.

This process for the production of testosterone (TS) has been developed to compete with current chemical synthesis procedures. The major obstruction in bringing this biotechnological process from laboratory to industrial scale production will be optimizing the production of TS from sterols in a single biotransformation step. The reversibility of 17β‐HSD enzymes and cell metabolic state are two most important determinants for improvement of this process. Attempts are being carried to design mutants of 17β‐HSD having improved substrate specificity and coenzyme requirements. The host cell's metabolic state should also be modified using carbon source supplements, determining adequate pH or varying the mode of substrate addition that will help in efficient reduction of AD to TS (Fig. 1). This will take time and is a very long process, but when successfully accomplished will replace the current procedure of chemical synthesis. This work also opens up the possibilities of producing TS analogues with better ability by genetic manipulations in the genes responsible for TS formation. The stable analogues could have a better shelf life and improved efficiency. Even though the work provides a proof of concept to start with, this is still a major achievement. This work has opened up possibilities of this model bacterium being used for the production of important pharmaceutical steroids using metabolic engineering approaches.

## Conflict of interest

None declared.

## References

[mbt212466-bib-0001] Bachman, E. , Travison, T.G. , Basaria, S. , Davda, M.N. , Guo, W. , Li, M. , *et al* (2014) Testosterone induces erythrocytosis via increased erythropoietin and suppressed hepcidin: evidence for a new erythropoietin/hemoglobin set point. J Gerontol A Biol Sci Med Sci 69: 725–735. 24158761 10.1093/gerona/glt154 PMC4022090

[mbt212466-bib-0002] Barrett‐Connor, E. (1992) Lower endogenous androgen levels and dyslipidemia in men with non‐insulin‐dependent diabetes mellitus. Ann Intern Med 117: 807–811. 1416554 10.7326/0003-4819-117-10-807

[mbt212466-bib-0003] Barrett‐Connor, E. , and Khaw, K.T. (1988) Endogenous sex hormones and cardiovascular disease in men. A prospective population‐based study. Circulation 78: 539–545. 3409497 10.1161/01.cir.78.3.539

[mbt212466-bib-0004] Batrinos, M.L. (2012) Testosterone and Aggressive Behavior in Man. Int J Endocrinol Metab 10: 563–568. 23843821 10.5812/ijem.3661 PMC3693622

[mbt212466-bib-0005] Campion, J.M. , and Maricic, M.J. (2003) Osteoporosis in men. Am Fam Physician 67: 1521–1526. 12722852

[mbt212466-bib-0006] Charney, W. , and Herzog, L. (1967) Microbial transformations of steroids. New York, London: Academic Press p, p. 728.

[mbt212466-bib-0007] Donova, M.V. , Egorova, O.V. , and Nikolayeva, V.M. (2005) Steroid 17β‐reduction by microorganism‐ a review. Process Biochem 40: 2253–2262.

[mbt212466-bib-0008] Dutta, R. , Roy, M. , and Singh, H. (1992) Metabolic blocks in the degradation of beta‐sitosterol by a plasmid‐cured strain of *Arthrobacter oxydans* . J Basic Microbiol 32: 167–176. 1512707 10.1002/jobm.3620320305

[mbt212466-bib-0009] Ercoli, A. , and Ruggierii, P.D. (1953) An improved method of preparing testosterone, dihydrotestosterone and some of their esters. J Am Chem Soc 75: 650–653.

[mbt212466-bib-0100] Fernández‐Cabezón, L. , Galán, B. , and García, L.J. (2016) Engineering *Mycobacterium smegmatis* for testosterone production. Microb Biotechnol (Accepted). 10.1111/1751-7915.12433 PMC5270716 27860310

[mbt212466-bib-0010] Fogal, S. , Bergantino, E. , Motterle, R. , Castellin, A. and Arvotti, A. (2013) Process for the preparation of testosterone. Patent US 2013/8592178B2.

[mbt212466-bib-0011] Griggs, R.C. , Kingston, W. , Jozefowicz, R.F. , Herr, B.E. , Forbes, G. , and Halliday, D. (1989) Effect of testosterone on muscle mass and muscle protein synthesis. J Appl Physiol 66: 498–503. 2917954 10.1152/jappl.1989.66.1.498

[mbt212466-bib-0012] Groman, E. , and Engel, L. (1977) Hydroxysteroid dehydrogenase of *Pseudomonas testosteroni* Separation of 17β‐hydroxysteroid dehydrogenase from 3(17)β‐hydroxysteroid dehydrogenase and comparison of the two enzymes. Biochim Biophysica Acta 485: 249–254. 10.1016/0005-2744(77)90161-9 411517

[mbt212466-bib-0013] Haffner, S.M. , Mykkänen, L. , Valdez, R.A. , and Katz, M.S. (1993) Relationship of sex hormones to lipids and lipoproteins in nondiabetic men. J Clin Endocrinol Metab 77: 1610–1615. 8263149 10.1210/jcem.77.6.8263149

[mbt212466-bib-0014] Hamilton, J.B. (1937) Treatment of sexual underdevelopment with synthetic male hormone substance. Endocrinology 21: 649–654.

[mbt212466-bib-0015] Kontoleon, P.E. , Anastasiou‐Nana, M.I. , Papapetrou, P.D. , Alexopoulos, G. , Ktenas, V. , Rapti, A.C. , *et al* (2003) Hormonal profile in patients with congestive heart failure. Int J Cardiol 87: 179–183. 12559538 10.1016/s0167-5273(02)00212-7

[mbt212466-bib-0016] Kratzik, C.W. , Schatzl, G. , Lunglmayr, G. , Rucklinger, E. , and Huber, J. (2005) The impact of age, body mass index and testosterone on erectile dysfunction. J Urol 174: 240–243. 15947646 10.1097/01.ju.0000162049.95483.51

[mbt212466-bib-0017] Kumar, R. , Dahiya, J. , Singh, D. , and Nigam, P. (2001) Biotransformation of cholesterol using *Lactobaccillus bulgaricus* in a glucose‐controlled bioreactor. Bioresour Technol 78: 209–211. 11333043 10.1016/s0960-8524(00)00174-7

[mbt212466-bib-0018] Liu, W.H. , and Lo, C.K. (1997) Production of testosterone from cholesterol using a single‐step microbial transformation of *Mycobacterium* sp. J Ind Microbiol Biotechnol 19: 269–272. 9439002 10.1038/sj.jim.2900456

[mbt212466-bib-0019] Liu, W.H. , Kuo, C.W. , Wu, K.L. , Lee, C.Y. , and Hsu, W.Y. (1994) Transformation of cholesterol to testosterone by *Mycobacterium* sp. J Ind Microbiol 13: 167–171.

[mbt212466-bib-0020] Mahato, S. , and Mukherjee, A. (1984) Microbial transformation of testosterone by *Aspergillus fumigatus* . J Steroid Biochem 21: 341–342. 6387279 10.1016/0022-4731(84)90289-9

[mbt212466-bib-0021] Matsumoto, A.M. (1990) Effects of chronic testosterone administration in normal men: safety and efficacy of high dosage testosterone and parallel dose‐dependent suppression of luteinizing hormone, follicle‐stimulating hormone, and sperm production. J Clin Endocrinol Metab 70: 282–287. 2104626 10.1210/jcem-70-1-282

[mbt212466-bib-0022] Moffat, S.D. , Zonderman, A.B. , Metter, E.J. , Kawas, C. , Blackman, M.R. , Harman, S.M. , and Resnick, S.M. (2004) Free testosterone and risk for Alzheimer disease in older men. Neurology 62: 188–193. 14745052 10.1212/wnl.62.2.188

[mbt212466-bib-0023] Morley, J.E. , and Perry, H.M.I.I.I. (2000) Androgen deficiency in aging men: role of testosterone replacement therapy. J Lab Clin Med 135: 370–378. 10811051 10.1067/mlc.2000.106455

[mbt212466-bib-0024] Phillips, G.B. , Jing, T.Y. , Resnick, L.M. , Barbagallo, M. , Laragh, J.H. , and Sealey, J.E. (1993) Sex hormones and hemostatic risk factors for coronary heart disease in men with hypertension. J Hypertens 11: 699–702. 8228187 10.1097/00004872-199307000-00003

[mbt212466-bib-0025] Plésiat, P. , Grandguillot, M. , Harayama, S. , Vragar, S. , and Michel‐Briand, Y. (1991) Cloning, sequencing and expression of the *Pseudomonas testosteroni* gene encoding 3‐oxosteroid δ1 ‐dehydrogenase. J Bacteriol 173: 7219–7227. 1657885 10.1128/jb.173.22.7219-7227.1991 PMC209228

[mbt212466-bib-0026] Svartberg, J. , von Muhlen, D. , Schirmer, H. , Barrett‐Connor, E. , Sundfjord, J. , and Jorde, R. (2004) Association of endogenous testosterone with blood pressure and left ventricular mass in men. The Tromsø Study. Eur J Endocrinol 150: 65–71. 14713281 10.1530/eje.0.1500065

[mbt212466-bib-0027] Tripathy, D. , Shah, P. , Lakshmy, R. , and Reddy, K.S. (1998) Effect of testosterone replacement on whole body glucose utilisation and other cardiovascular risk factors in males with idiopathic hypogonadotrophic hypogonadism. Horm Metab Res 30: 642–645. 9851674 10.1055/s-2007-978950

[mbt212466-bib-0028] Uwajima, T. , Yagi, H. , Nakamura, S. , and Terada, O. (1973) Isolation and crystallization of extracellular 3β‐hydroxysteroid oxidase *of Brevibacterium sterolicum* nov. sp. Agric Biol Chem 37: 2345–2350.

[mbt212466-bib-0029] Wix, G. , Buki, K. , Tomorken, E. , and Ambrus, G. (1968) Inhibition of steroid nucleus degradation in mycobacterial transformation. Steroids 11: 401–413. 5642330 10.1016/s0039-128x(68)80150-3

